# The glucose tolerance test in mice: Sex, drugs and protocol

**DOI:** 10.1111/dom.14811

**Published:** 2022-07-25

**Authors:** Matilda R. Kennard, Manasi Nandi, Sarah Chapple, Aileen J. King

**Affiliations:** ^1^ Department of Diabetes King's College London London UK; ^2^ Institute of Pharmaceutical Science King's College London London UK; ^3^ School of Cardiovascular Medicine & Sciences King's College London London UK

**Keywords:** continuous glucose monitoring (CGM), mouse model, animal, pharmacology, antidiabetic drug, type 2 diabetes

## Abstract

**Aim:**

To establish the impact of sex, dosing route, fasting duration and acute habituation stress on glucose tolerance test (GTT) measurements used in the preclinical evaluation of potential glucose‐modulating therapeutics.

**Methods:**

Adult male and female C57Bl/6J mice, implanted with HD‐XG glucose telemetry devices, were fasted for 16 hours or 6 hours following acute habituation stress due to whole cage change, cage change with retention of used bedding or no cage change prior to intraperitoneal (IP) GTTs. To evaluate protocol refinement and sex on the ability of the GTT to detect drug effects, we administered 250 mg/kg oral metformin or 10 nmol/kg IP exendin‐4 using optimized protocols.

**Results:**

Female mice were less sensitive to human intervention when initiating fasting. Following a 6‐hour fast, retention of bedding whilst changing the cage base promotes quicker stabilization of basal blood glucose in both sexes. Prolonged fasting for 16 hours resulted in an exaggerated GTT response but induced pronounced basal hypoglycaemia. Following GTT protocol optimization the effect of exendin‐4 and metformin was equivalent in both sexes, with females showing a more modest but more reproducible GTT response.

**Conclusions:**

Variations in GTT protocol have profound effects on glucose homeostasis. Protocol refinement and/or the use of females still allows for detection of drug effects, providing evidence that more severe phenotypes are not an essential prerequisite when characterizing/validating new drugs.

## 1 INTRODUCTION

Animal models play an important role in preclinical diabetes research, with glucose tolerance tests (GTTs) being a popular tool for detecting impaired glucose homeostasis.^1^, ^2^ GTTs are also often used to test new antidiabetic treatments on stimulated blood glucose concentrations^3^ and are one of the most commonly undertaken experiments in metabolic research.

Female mice are often excluded from diabetes research as they become less glucose‐intolerant and insulin‐resistant following induction of the disease in almost all models.^4^, ^5^, ^6^, ^7^ Indeed, this relative lack of phenotype is commonly believed to impede their usefulness in drug efficacy studies, creating preclinical bias and potentially impeding translation to a heterogenous clinical population. Their perceived increased variability in blood glucose concentrations across the oestrous cycle may also explain a reluctance to study female mice.^8^, ^9^ The impact of sex and the oestrous cycle on blood glucose variability in the GTT has not previously been studied in detail, despite growing emphasis on the need to consider sex as a biological variable in preclinical studies.^9^, ^10^, ^11^

Despite their importance, GTTs are poorly standardized in preclinical settings, with laboratory‐to‐laboratory variation in almost all aspects of the protocol. Fasting is undertaken prior to GTTs to minimize the influence of variability in food intake on basal blood glucose concentrations.^12^, ^13^ However, there is no standard fasting duration despite evidence that different fast lengths can substantially impact both welfare and blood glucose concentrations.^1^, ^13^ Indeed, overnight fasting is associated with significant welfare implications including weight loss and hypoglycaemia,^13^, ^14^, ^15^, ^16^ but is still commonly used.

As fasting is undertaken to eliminate the influence of food, some researchers may change their mice's cages at the start of the fast to ensure that no food remnants are present in the bottom, although the technical aspects of this protocol are not usually described in methods sections. Indeed, it has previously been shown that 18% to 36% of food is dropped into the bottom of the cage during feeding^17^ and this spillage can account for up to 40% of food consumption.^18^, ^19^ Hence, not changing the cage may limit the efficacy of fasting. However, whole cage changes are associated with various stress responses in C57Bl/6J mice including increased corticosterone, blood pressure and anxiety‐like behaviours.^20^ A potential balance between these two cage‐change methods involves changing the base of the cage where most food accumulates but retaining bedding from the previous cage in order to maintain familiar smells. This method has previously been shown to reduce stress responses^20^ although the impact of this intervention on GTT outcomes has not previously been documented.

Finally, the act of administering glucose could also affect GTT outcome beyond that of well‐established differences in routes of administration due to the incretin effect.^1^, ^21^, ^22^, ^23^, ^24^, ^25^, ^26^ In most cases, animal restraint is required for glucose administration by either intraperitoneal (IP) injection or oral gavage, which could lead to stress responses and subsequent changes in glucose homeostasis.^27^ However, mice can be trained relatively easily to consume a glucose gel. This allows for unrestrained glucose administration, which would be regarded as further refinement, but may impact outcomes due to both the potential for reduced stress and enhanced incretin responses when compared to IP injection and oral gavage.^28^, ^29^, ^30^

Whilst previous research has addressed the lack of GTT standardization in metabolic research, there is an absence of comprehensive data exploring the effect of different experimental and physiological variables on blood glucose concentrations.^1^, ^14^ This information is essential to tackle biases in experimental design (such as excluding females) and to allow researchers to understand how minor variations in protocols could affect outcome. We have used continuous glucose monitoring technology^31^ to better understand the impact of various researcher interventions involved in initiating the GTT, with a particular focus on fasting protocols. This allowed us to measure blood glucose concentrations continuously in unrestrained mice both during and after minor interventions (eg, initiating fasting) even after the researcher had left the room. Consequently, we were able to obtain detailed information on the acute impact of interactions including the magnitude and longevity of blood glucose peaks. We used this information to identify which researcher interventions had the least impact on the mice and then sought to determine whether the most refined procedures and the inclusion of female mice altered the ability to detect clinically relevant drug effects during GTTs.

## 2 MATERIALS AND METHODS

### 2.1 Animals

Seven male and seven female C57Bl/6J mice (aged 8‐10 weeks on arrival; Charles River, Tranent, UK) were implanted with HD‐XG glucose telemetry probes (Data Science International, St Paul, Minnesota) after 2 weeks of acclimatization and baseline measurements of weight and blood glucose concentration via glucometer (StatStrip Xpress; NovaBiomedical, Waltham, Massachusetts). These mice were used to understand how researcher intervention impacted blood glucose concentrations during the experiment. At the time of experimentation, the mice were aged between 10 and 14 weeks and weighed between 25.4 ± 0.8 g and 29.2 ± 1.0 g (males) and 18.5 ± 0.5 g and 21.9 ± 0.8 g (females).

The mice were separated into three cohorts: males, females in proestrous‐oestrous (P‐E) and females in metoestrous‐dioestrous (M‐D). To avoid the intervention of vaginal swabbing confounding the GTT results, oestrous swabs were obtained at the end of experimentation and therefore the number of mice in each oestrous stage was random and varied for each experiment.

A further 10 non‐telemetered male (10‐12 weeks, 26.9 ± 0.7 g) and female (10‐12 weeks, 20.4 ± 0.5 g) C57Bl/6J mice were then used to consider whether voluntary consumption of oral glucose gels altered glucose responses to oral gavage. Finally, a further eight male (10‐12 weeks, 27.2 ± 1.0 g) and female (10‐12 weeks, 20.9 ± 0.7 g) mice were studied to investigate our most refined protocols in normal nonoperated mice, to ensure validity of the results in normal laboratory settings.

All mice were housed in a controlled environment at 22°C with free access to standard Rodent Diet 20 chow (Picolab, London, UK) and water, and maintained in 7:00 am to 7:00 pm light: dark cycles. Mice were housed in cages with a nonlittermate, nonsurgical “buddy” of the same sex, with a maximum of one telemetry mouse per cage. Mice were habituated to their buddy for at least 2 weeks prior to surgery or experimentation as our previous experience indicated this reduced fighting between males. Cameras on the cages allowed behaviour to be monitored, and fighting between male mice was extremely rare. The sporadic fighting observed only had very transient effects on blood glucose concentrations and did not coincide with our experiments. Experimentation was undertaken in specialized telemetry holding rooms without the need to move the mice.

All experiments were approved by the institution's welfare and ethics committee and undertaken in accordance with the UK Animals (Scientific Procedures) Act 1986 with 2012 amendments.

### 2.2 Surgical implantation of glucose telemetry probes and recovery

Mice were implanted with HD‐XG glucose telemetry devices in the aortic arch under general anaesthesia (Supplementary Information). Subcutaneous 4 mg/kg carprofen (Carprieve; Centaur, London, UK) in sterile saline (AquaPharm; Centaur) was administered immediately prior to surgery and 24 hours after surgery. Experiments were commenced 1 week after surgery to ensure full surgical recovery^31^ and habituation to the probe. Probe calibration was carried out in line with manufacturer's guidelines, as previously described in detail.^31^

### 2.3 Oestrous swabbing and staining

Vaginal smears were obtained for 10 consecutive days to determine oestrous cycling in each female mouse and, where relevant, at the end of each GTT. Smears were placed onto slides and stained with methylene blue for 12 minutes (Sigma‐Aldrich, Gillingham, UK). Mice were staged based on the relative proportions of nucleated epithelium, cornified epithelium and leukocytes, as observed with a light microscope.^32^ Mice were separated into P‐E status, when oestrogen is normally high, and M‐D status, when oestrogen is normally low.

### 2.4 Effect of cage change method and fast length on blood glucose concentration

Telemetered mice were fasted for 6 hours (from 9:00 am‐3:00 pm) with a whole cage change, bedding retention cage change or no cage change at the start of the fast (Table 1 and Supplementary Information). Mice were also fasted for 16 hours (commencing at 5:00 pm) with a bedding retention cage change only.

**TABLE 1:** Cage changing methods used at the start of 6‐hour daytime and 16‐hour overnight fasting

Fast length	Time of fast	Cage change	Food removed	Mice handled	New cage and wood chippings	Retention of used bedding and enrichment
6 hours	9:00 am	Whole cage change	✓	✓	✓	✗
		Bedding retention cage change	✓	✓	✓	✓
		No cage change	✓	✗	✗	✓
16 hours	5:00 pm	Bedding retention cage change	✓	✓	✓	✓

Ticks and crosses represent whether or not this occurred for that particular method.

### 2.5 Effect of cage change method and fast length on GTT outcome

At the end of 6‐hour or 16‐hour fasting, the mice were weighed and a baseline blood glucose concentration was measured via glucometer (Nova Biomedical). Mice were then administered 2 g/kg glucose (Sigma‐Aldrich) in sterile saline (AquaPharm; Centaur) via IP injection. Repeat blood glucose concentrations were measured at 15, 30, 60, 90 and 120 minutes after glucose administration. Telemetry probes simultaneously measured blood glucose concentrations every 10 seconds for the duration of the GTT.

### 2.6 Effect of voluntary oral route of glucose administration

Based on results from the above, refined procedures were used to determine whether further refinement of oral glucose administration was possible using voluntarily consumed gels (Supplementary Information). Mice were separated from their buddies whilst gels were administered using wooden cage separators to ensure that each mouse ate its own gel. Mice were trained to eat the glucose gels over 1 week, as previously described in detail.^29^ Any mouse that ate less than 90% of the gel within this 60‐second period was excluded from the oral gel study (corresponding to 6% of results).

Once trained to voluntarily eat oral glucose gels, mice were fasted for 6 hours (9:00 am to 3:00 pm) with a bedding retention cage change. After the initial blood glucose concentrations were measured, mice were either administered 2 g/kg glucose via oral gavage or presented with the gel. The GTT then proceeded as described above.

### 2.7 Effect of drugs using refined procedures

Based on the data generated, we concluded that the most refined method with least variable fasting and GTT data was a 6‐hour fast commencing at 9:00 am after a bedding retention cage change, followed by glucose administration via voluntary consumption of oral gels (Supplementary Information). Using these refined procedures, we studied whether this protocol could be used to robustly assess pharmacological interventions. Therefore, 30 minutes prior to the end of the fast, a baseline blood glucose concentration was obtained simultaneously via glucometer and telemetry. The mice were then administered either 250 mg/kg metformin (Sigma‐Aldrich) via oral gels using sugar‐free sucralose sweetener as control, or 10 nmol/kg exendin‐4 (Sigma‐Aldrich) via IP injection using saline as control to mimic the clinically relevant routes of administration. Gels were administered in the same way as for glucose gels. Thirty minutes after drug or control administration, 2 g/kg glucose was administered via voluntary ingestion of glucose gels, as described above. Telemetry probes also obtained blood glucose concentrations every 10 seconds for the duration of the GTT.

### 2.8 Statistical methods

Changes in blood glucose concentrations due to researcher intervention and fasting were quantified by area under the curve (AUC), maximal and minimal blood glucose concentrations and time spent above or below baseline. Furthermore, time taken to reach blood glucose concentrations below fed levels was measured. In these experiments, two‐way ANOVA with a Holm‐Sidak post hoc test was undertaken to investigate the effect of researcher intervention (cage change/fast length) and sex of the animal studied (males, females in P‐E and females in M‐D). For GTT data, two‐way repeated‐measures ANOVA with Holm‐Sidak post hoc test was used to detect differences in different experimental groups (eg, cage change/fast length/experimental drug) over time. In addition, AUC was analysed by two‐way ANOVA with a Holm‐Sidak post hoc test.

To ascertain whether different numbers of male and female mice were required for testing drugs, sample size calculations were undertaken for drug versus control GTTs using the difference in means and standard deviations between AUC_150_ for both drugs versus their respective controls and the 15‐minute post‐glucose blood glucose concentrations. Criteria were set at a power of 0.90 and alpha value of 0.01.

All graphical and statistical analyses were undertaken using SigmaPlot 14.0. Data were normally distributed and are presented as mean ± standard error of the mean (SEM), with *P* values < 0.05 taken to represent statistical significance.

## 3 RESULTS

### 3.1 Fasting and cage changing cause a biphasic response in blood glucose concentrations

Continuous glucose monitoring data collected over 36 hours showed the impact of the whole GTT process, including the start of fast, the impact of fast length, the extent of the glucose peak, and recovery from the procedure (Figure S1). Blood glucose concentrations for both males and females fasted for 6 hours and 16 hours suggested a biphasic response to fasting prior to a GTT with 1) initial increases in blood glucose following human intervention to initiate the fast; and 2) prolonged reductions in glucose due to removal of food. Therefore, fasting data were separated and analysed from 0 to 120 minutes and 120 to 330 minutes (6‐hour fasts) or 120 to 900 minutes (16‐hour fasts) to reflect these different phases.

### 3.2 Blood glucose concentrations initially increase when cages are interfered with for fasting

**FIGURE 1:**
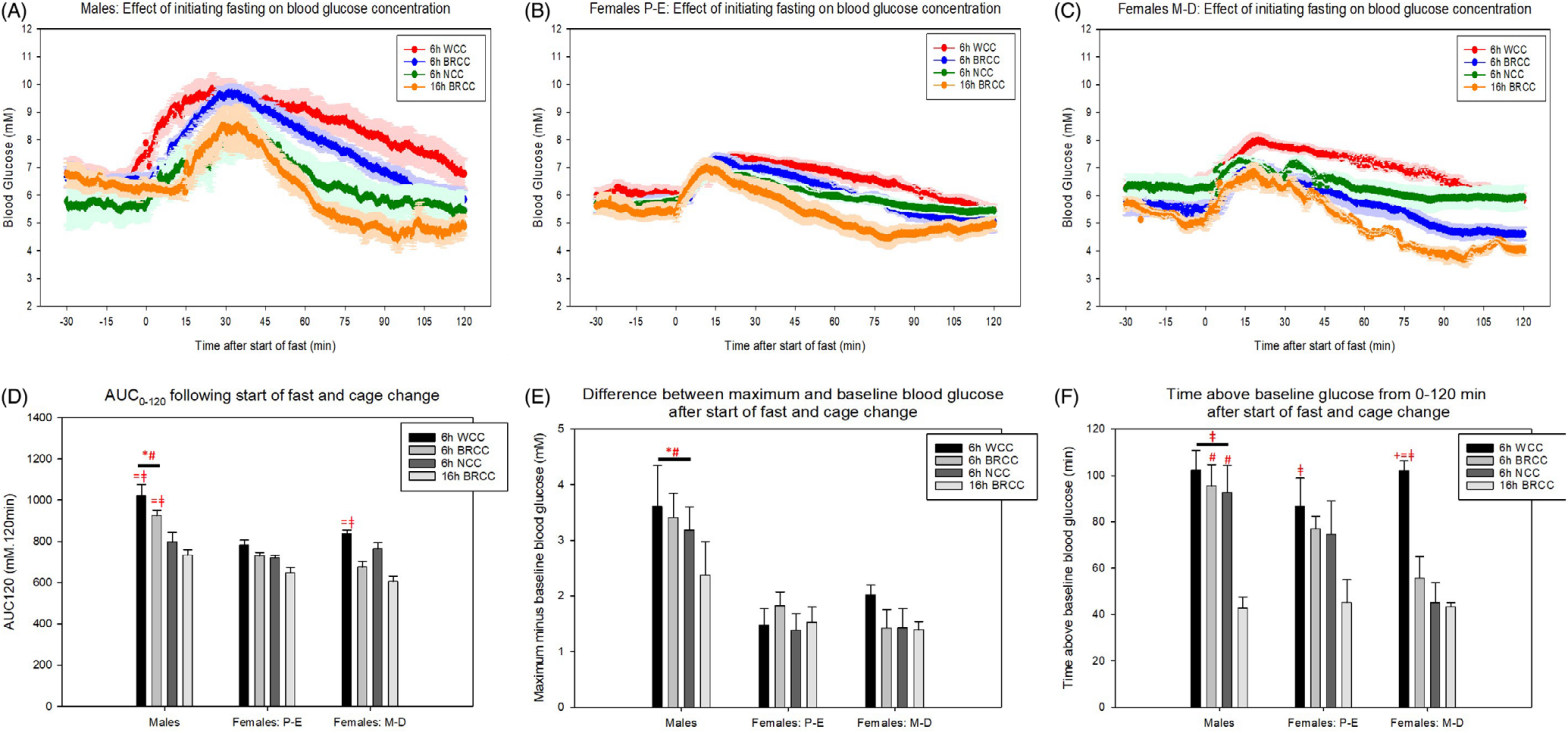
The initial effects of 6‐hour and 16‐hour fasting on blood glucose concentrations from 30 minutes pre‐intervention to 120 minutes post‐intervention in (A) males, (B) females in proestrous‐oestrous (P‐E) and (C) females in metoestrous‐dioestrous (M‐D). (D) Area under the curve (AUC)_120_ for graphs (A‐C). (E) Difference between maximum blood glucose reached from 0 to 120 minutes following intervention and pre‐intervention baseline. (F) Time spent above pre‐intervention blood glucose concentrations from 0 to 120 minutes post‐intervention. Pre‐intervention glucose was quantified as the average glucose between −30 and −15 minutes. * and # represent a significant difference compared to females in P‐E and M‐D, respectively. ~, +, = and ⱡ represent a significant difference compared to 6 hours with whole cage change (WCC), 6 hours with bedding retention cage change (BRCC), 6 hours with no cage change (NCC) and 16 hours with BRCC, respectively (*P* < 0.05, two‐way ANOVA with Holm‐Sidak post hoc tests). Data are mean ± SEM (n = 7 for males, n = 5‐7 for females in P‐E and n = 4‐7 for females in M‐D)

In both males and females, initiating fasting at 9:00 am (for 6‐hour daytime fasts) produced a transient increase in blood glucose concentration irrespective of cage change method or oestrous stage, with blood glucose being significantly higher than pre‐intervention levels for at least 80 minutes in males and 45 minutes in females (Figure 1A‐C,F). Initiating fasting at 5:00 pm (for 16‐hour overnight fasts) with a bedding retention cage change (BRCC) increased blood glucose for up to 45 minutes in males and 30 minutes in females.

The magnitude of change in blood glucose concentrations was larger in males regardless of cage change method or time of fast initiation, with blood glucose concentrations increasing by an average of 51.9% ± 9.1% in males compared to 23.6% ± 1.4% and 27.5% ± 3.0% in females in P‐E and M‐D, respectively. This was supported by significantly higher AUC_0–120_ and difference between maximum and pre‐intervention glucose in male mice (Figure 1D,E). Time spent above pre‐intervention glucose was also significantly higher in male mice but only when fasting commenced at 9:00 am (Figure 1E).

### 3.3 Blood glucose concentration increases are most pronounced when the whole cage is changed

The AUC_0–120_ was highest following whole cage changes in all cohorts regardless of sex or oestrous stage (Figure 1D). This appeared to be attributable to a combination of both prolonged responses and increased magnitude of response with whole cage changes (Figure 1E,F). In all cohorts, 9:00 am fasts produced a more prolonged initial response than 5:00 pm fasts (Figure 1F).

### 3.4 Six‐hour fasting is least effective when the cage is not changed, with 16‐hour fasting potentiating blood glucose reductions but to a point of hypoglycaemia

**FIGURE 2:**
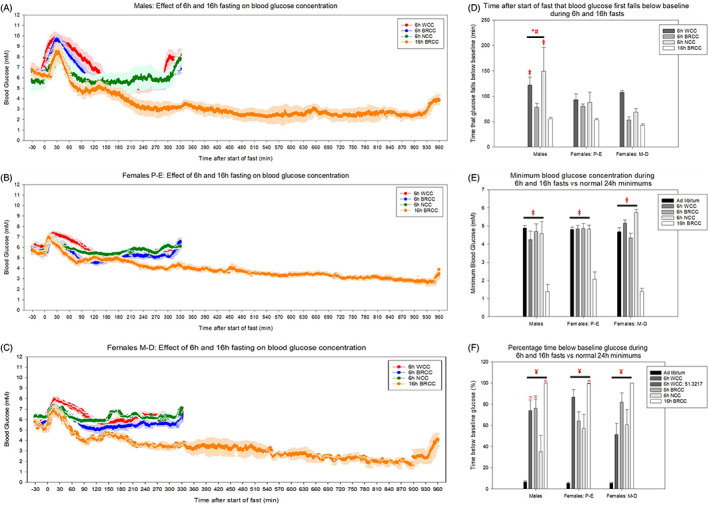
The prolonged effects of 6‐hour (6h) and 16‐hour (16h) fasting on blood glucose concentrations from 30 minutes pre‐intervention to 330 minutes and 960 minutes post‐intervention for 6‐hour and 16‐hour fasts, respectively, in (A) males, (B) females in proestrous‐oestrous (P‐E) and (C) females in metoestrous‐dioestrous (M‐D). (D) Time after start of fast that blood glucose fell below pre‐intervention concentrations for 5 consecutive minutes. (E) Minimum blood glucose concentration reached during fasting compared to normal 24‐hour minimum glucose concentrations with ad libitum food. (F) Percentage of time spent below pre‐intervention blood glucose concentrations from 0 to 330 or 0 to 960 minutes post‐intervention compared to normal ad libitum conditions. Pre‐intervention glucose was quantified as the average glucose between −30 and −15 minutes. * and # represent a significant difference compared to females in P‐E and M‐D, respectively. ~, +, =, ¥ and ⱡ represent a significant difference compared to 6 hours with whole cage change (WCC), 6 hours with bedding retention cage change (BRCC), 6 hours with no cage change (NCC), ad libitum conditions and 16 hours with BRCC, respectively (*P* < 0.05, two‐way ANOVA with Holm‐Sidak post hoc tests). Data are mean ± SEM (n = 7 for males, n = 5‐7 for females in P‐E and n = 4‐7 for females in M‐D)

As described above, the initial part of the fast was characterized by increased blood glucose concentrations in accordance with previous data.^33^ However, the aim of fasting is to reduce blood glucose concentrations to a basal level which should be lower than in the fed scenario. Overall, male mice took longer than female mice to reach blood glucose concentrations below normal fed concentrations (Figure 2A‐D). In both sexes, the quickest achievement of below‐fed blood glucose concentrations was following 16‐hour fasting commencing at 5:00 pm (Figure 2D). Furthermore, for 6‐hour fasting starting at 9:00 am, time taken for this effect was quickest when the bedding was retained during the cage change.

To control for normal fluctuations in blood glucose concentrations, fasted mice were compared to mice fed ad libitum. Minimum blood glucose during 6‐hour fasts did not fall below normal 24‐hour minimum concentrations in the fed state regardless of cage change method (Figure 2E). Conversely, 16‐hour overnight fasting resulted in lower minimum glucose concentrations for all cohorts when compared to both normal 24‐hour minimums and minimums achieved during 6‐hour fasting, regardless of cage change method. This minimum occurred at 12.2 ± 1.2 and 14.0 ± 0.7 hours in males and females, respectively. Some mice became hypoglycaemic, with blood glucose concentrations <2.8 mM (the lower limit of accuracy for glucose telemetry devices).

Under normal ad libitum conditions, blood glucose concentrations only fell below pre‐intervention blood glucose concentrations ~5% of the time, whereas in fasted mice this ranged from 30% to 100%. In all groups, mice fasted for 16 hours spent the highest percentage of time below pre‐intervention levels. In 6‐hour fasted mice, not changing the cage resulted in the least time spent below pre‐intervention levels, particularly in males (Figure 2F).

### 3.5 Cage change method and fast length alters glucose tolerance test outcome

**FIGURE 3:**
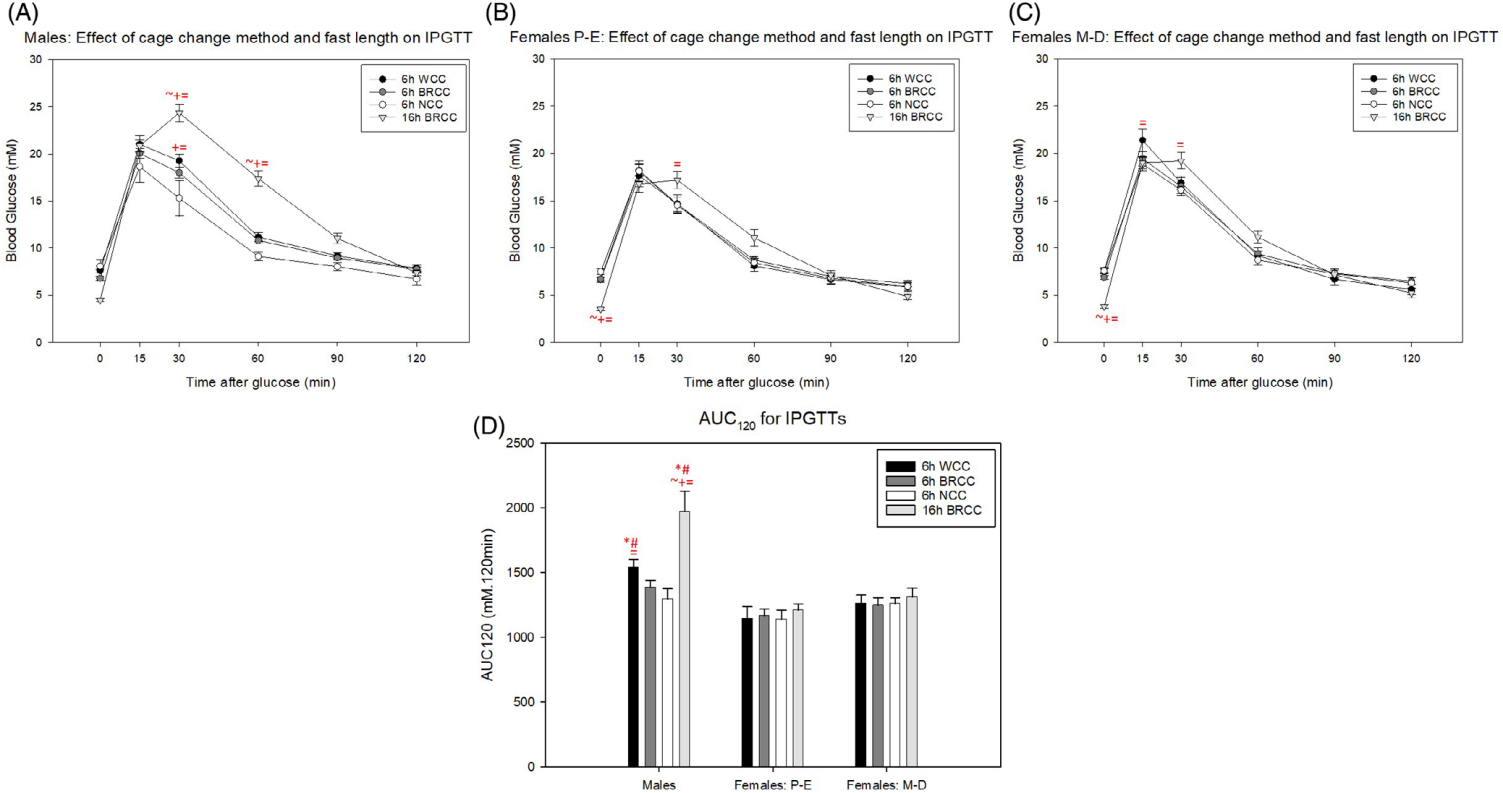
Effect of 6‐hour (6h) and 16‐hour (16h) fasts with different cage change methods on intraperitoneal glucose tolerance test (IPGTT) outcome in (A) males, (B) females in proestrous‐oestrous (P‐E) and (C) females in metoestrous‐dioestrous (M‐D). (D) Area under the curve (AUC)_120_ for graphs A‐C. * and # represent a significant difference compared to females in P‐E and M‐D, respectively. ~, + and = represent a significant difference compared to 6 hours with whole cage change (WCC), 6 hours with bedding retention cage change (BRCC) and 6 hours with no cage change (NCC), respectively (*P* < 0.05, two‐way repeated‐measures ANOVA with Holm‐Sidak post hoc tests). Data are mean ± SEM (n = 7 for males, females in P‐E and females in M‐D)

Intraperitoneal glucose administration increased blood glucose concentration at 15 to 30 minutes in all mice regardless of cage change method, fast length, sex or oestrous stage. However, 16‐hour fasts were associated with impaired glucose tolerance in all cohorts, with elevated blood glucose concentrations at 30 to 60 minutes in males and 30 minutes in females both in P‐E and in M‐D (Figure 3A‐C). Six‐hour fasted glucose tolerance was also worsened when the whole cage was changed in males and females in M‐D with increased blood glucose at 30 and 15 minutes, respectively.

Overall, male mice were more glucose‐intolerant than females, regardless of oestrous stage, with higher AUC_120_ values. However, this was exaggerated when the whole cage was changed at the start of 6‐hour fasting or when mice were fasted overnight (Figure 3D).

### 3.6 Oral gels provide further refinement of GTT protocol

**FIGURE 4:**
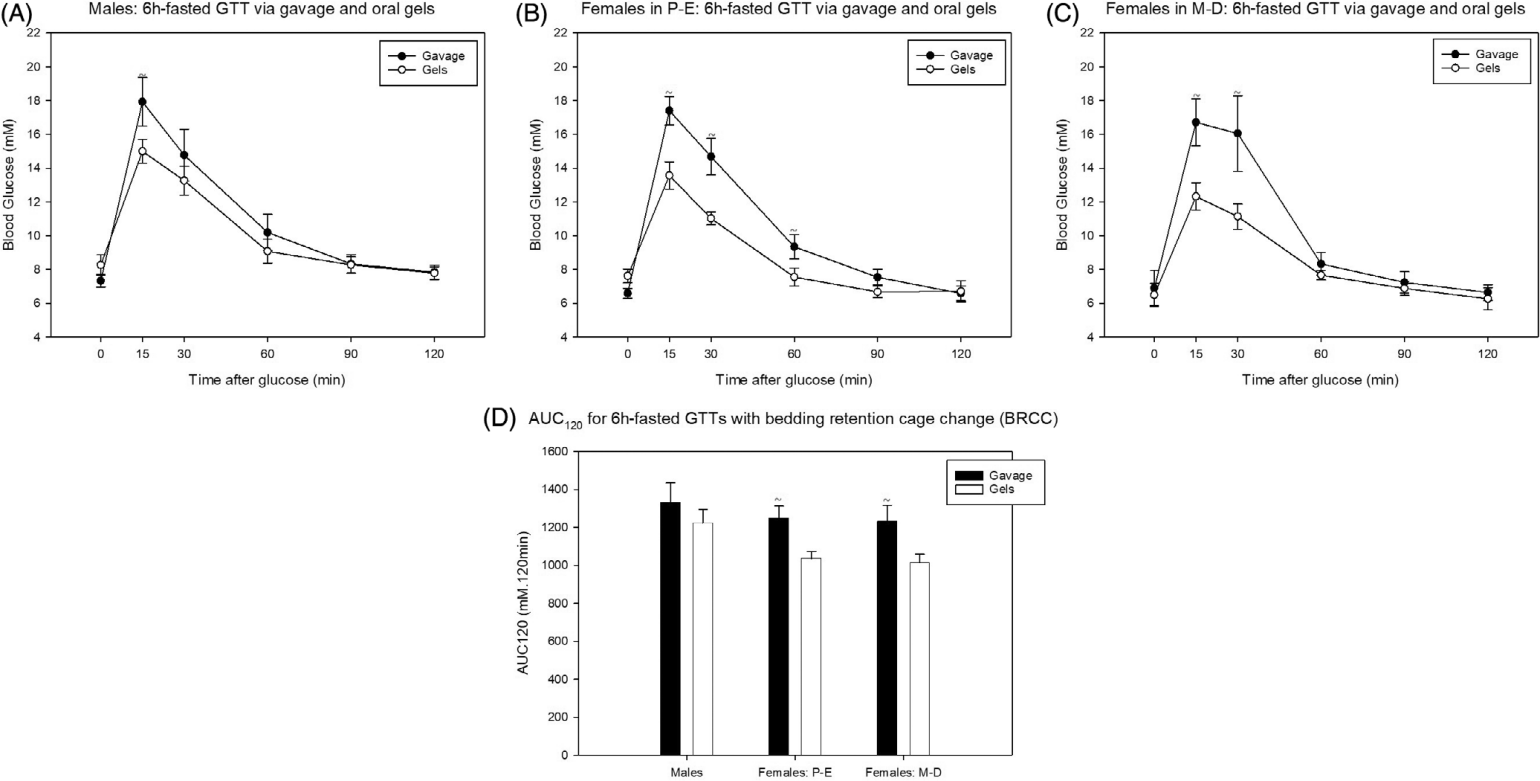
Effect of oral gavage and oral glucose gels on 6‐hour (6h)‐fasted intraperitoneal glucose tolerance test (IPGTT) outcome following bedding retention cage changes (BRCC) in (A) males; (B) females in proestrous‐oestrous (P‐E); and (C) females in metoestrous‐dioestrous (M‐D). (D) Area under the curve (AUC)_120_ for graphs (A‐C). * and # represent a significant difference compared to females in P‐E and M‐D, respectively. ~ represents a significant difference compared to oral glucose gels (*P* < 0.05, two‐way repeated‐measures ANOVA (A–C) and two‐way ANOVA) (D) with Holm‐Sidak post hoc tests). Data are mean ± SEM (n = 5 for males, females in P‐E and females in M‐D)

The experiments described above indicated that 1) 16‐hour fasts cause pronounced hypoglycaemia (blood glucose concentrations <2.8 mM); 2) 6‐hour fasts with whole cage changes increase initial stress responses; and 3) 6‐hour fasts with no cage change are least effective at swiftly reducing fasted blood glucose. Therefore, 6‐hour fasts with bedding retention cage change could be regarded as the most refined protocols whilst ensuring hepatic control of glucose homeostasis.^14^, ^34^, ^35^, ^36^ To further refine the protocol by reducing researcher intervention, we compared the effect of voluntary ingestion of gels and oral gavage of glucose. Both oral gels and gavage increased blood glucose at 15 to 30 minutes in females and 15 minutes in males (Figure 4A‐C). However, gavage produced a higher magnitude of blood glucose increase at these time points. Furthermore, the AUC_0–120_ was significantly higher for gavage versus gels in females, irrespective of oestrous cycling (Figure 4D).

### 3.7 The most refined protocol still allows for detection of drug effects

The experiments above indicated that more severe interventions (whole vs. bedding retention cage change, 16‐hour vs. 6‐hour fast, oral gavage vs. gel) produced higher blood glucose concentrations during a GTT. Therefore, to determine whether the most refined procedures could still detect glucose‐lowering effects of clinically relevant drugs, mice were fasted for 6 hours with bedding retention cage change before administration of exendin‐4 or metformin 30 minutes prior to an oral gel GTT.

**FIGURE 5:**
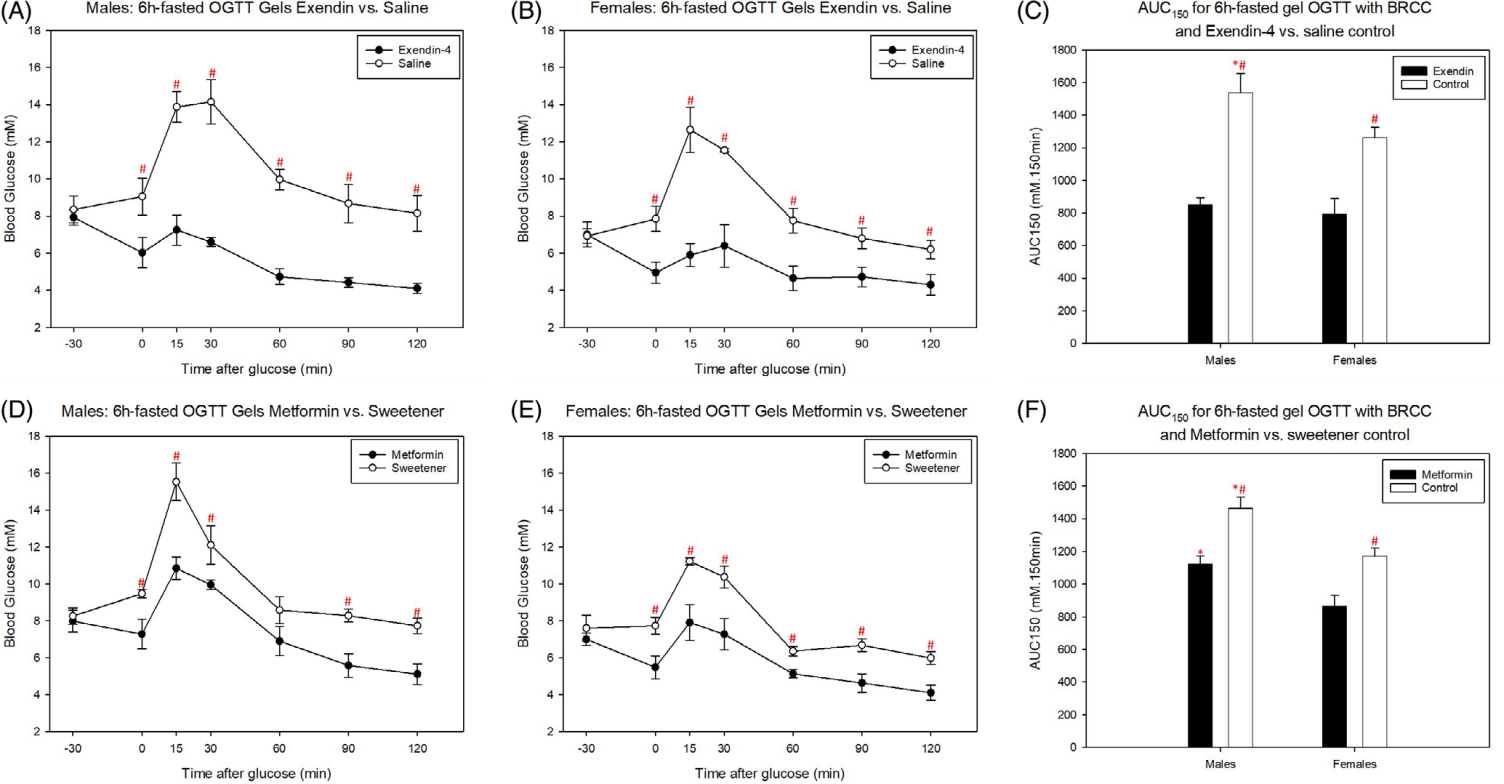
Effect of oral metformin and intraperitoneal (IP) exendin‐4 on 6‐hour (6h)‐fasted IP glucose tolerance test (GTT) outcome following bedding retention cage changes (BRCC). (A, B) IP exendin‐4 vs IP saline control in (A) males and (B) females. (D, E) Oral metformin versus sweetener gel control in (D) males and (E) females. (C and F) Area under the curve (AUC)_120_ for graphs (A, B) and (D, E), respectively. * and # represent a significant difference compared to females and control, respectively (*P* < 0.05, two‐way ANOVA with Holm‐Sidak post hoc tests). Data are mean ± SEM (n = 4 for males and females)

Both metformin and exendin‐4 significantly improved glucose tolerance in both males and females, with blood glucose at all time points after glucose administration being significantly higher for control‐treated versus drug‐treated mice (Figure 5A,B,D,E). This was supported by higher AUC_150_ values in control‐treated male and female mice (Figure 5C,F).

The difference in means between AUC_150_ values and 15‐minute blood glucose concentrations for drug‐treated versus control‐treated mice tended to be higher in males compared to females (Table 2). However, the standard deviation for these variables also tended to be higher in males. Consequently, sample size calculations with a desired power of 0.90 and alpha value of 0.01 revealed that the same number of mice would be required to detect a statistical difference between exendin or metformin versus controls in both sexes.

**TABLE 2:** Sample size calculations for 250 mg/kg oral metformin and 10 nmol/kg intraperitoneal exendin‐4 versus control glucose tolerance tests

AUC_150_	Difference in means, mM.120 min	Standard deviation, mM.120 min	Sample size
**Exendin‐4 vs. saline control**			
Males	689.4	183.0	4
Females	465.0	133.8	4
**Metformin vs. sweetener control**			
Males	342.8	124.9	6
Females	305.6	119.7	6
**15 min post‐glucose concentration**	**Difference in means (mM)**	**Expected standard deviation (mM)**	**Sample size**
**Exendin‐4 vs. saline control**			
Males	6.63	1.85	4
Females	5.75	1.50	4
**Metformin vs sweetener control**			
Males	4.68	1.61	5
Females	3.33	1.17	5

Calculations were undertaken using the difference in means and standard deviations for the area under the curve (AUC)_150_ for metformin vs. sweetener control and exendin‐4 vs. saline control and 15 minutes post‐glucose glucometer blood glucose concentration in both males and females.

## 4 DISCUSSION

Despite being one of the most commonly used tools in metabolic research, GTTs are poorly standardized. Researcher intervention cannot be completely avoided in a GTT and it is clear that even minor interventions contribute to increases in blood glucose concentrations.^33^ However, some interventions undertaken as part of the GTT protocol (eg, different fasting protocols) may be particularly stressful to mice^13^, ^14^, ^20^ which we hypothesized could further increase blood glucose concentrations and potentiate glucose intolerance.^37^, ^38^, ^39^, ^40^, ^41^, ^42^ Indeed, the initial impact of researcher intervention (increased blood glucose concentrations) was seen for the first 1‐2 hours of the fast in all our protocols, including when the mice were not handled, with the only researcher intervention involving removing the food from the lid of the cage. However, a whole cage change induced the most pronounced effect, which was evidenced by higher and more prolonged increases in blood glucose concentrations at the start of the fast as well as an impairment of glucose tolerance in male mice 6 hours later. This indicates that a detail as small as how the fast was initiated can affect GTT results. It may seem that the obvious answer is to avoid the cage change completely, and indeed animals that remained in their cage had the most subdued response at the start of the fast. However, fasting efficacy was reduced when the mice remained in their original cage with more variable, less pronounced and less timely glucose reductions. This was most likely attributable to food remnants on the cage bottom, as food spillage while feeding has previously been shown to be considerable.^17^, ^18^, ^19^ We suggest that changing the cage but retaining the original bedding is the best compromise between efficient fasting and reduced stress responses. With regard to the stark effect of a whole cage change, researchers should also consider that husbandry cage changes could impact results and this should be taken into account if an experimental series is carried out over several days.

It has been suggested that 4‐ to 6‐hour daytime fasting is sufficient to reduce blood glucose concentration, minimize gastric content and ensure gastric clearance of food consumed in the dark phase.^34^, ^35^, ^36^, ^52^ Despite this, the overnight fast is still widely used by researchers, with a review of the literature using the key phrase “glucose tolerance test and mouse” indicating that 60% of 353 studies between 2018 and 2019 still fasted mice overnight. In our study, overnight fasting was associated with marked hypoglycaemia, with blood glucose concentrations consistently falling to <2.8 mM at approximately 13 hours after fast initiation. A substantial lowering of blood glucose concentrations after fasting has previously been reported using glucometers^13^ but the extent of hypoglycaemia was probably underestimated as blood glucose is lowest a few hours before the end of the fast which is a time at which it not normally measured by researchers. In addition, handling the animal acutely increases blood glucose concentrations, thus masking the extent of hypoglycaemia.^33^ By monitoring blood glucose concentrations remotely, we have shown the extent of overnight‐fasted hypoglycaemia which is a clear welfare concern that was not observed with 6‐hour daytime fasting. In line with the results of the whole cage change, the excess stress of an overnight fast appears to impact the outcome of the GTT, with overnight‐fasted animals showing impaired glucose tolerance. Therefore, we would recommend avoiding overnight fasting unless clear justification is provided and, if a whole cage change is to be carried out, the simple refinement of retaining original bedding should be considered. It is worth noting that male mice were more sensitive to these interventions than females, with a higher magnitude and duration of blood glucose increases regardless of cage change method, fast length or oestrous stage, as previously seen with regards to other researcher interventions.^33^ Overall, these results challenge the concept that the use of female mice should be avoided due to increased variability due to the oestrous cycle.^5^, ^6^

Another reason researchers may predominately study male mice in GTTs is that females are more glucose‐tolerant than males in both the basal and glucose‐intolerant state.^42^, ^43^, ^44^ In line with this, female mice in our study were more glucose‐tolerant than male mice. The stage of oestrous had negligible effects on the GTT outcome, although it is worth pointing out that in mice at M‐D stage, whole cage changing led to increased blood glucose concentrations during the first 90 minutes of the fast when compared to other methods of initiating the fast. In addition, these mice showed a higher peak at 15 minutes in the GTT. In female mice that had been fasted overnight, both M‐D and P‐E groups of mice had an elevated peak at 30 minutes during the GTT. Although these impacts were minor compared to those seen in male mice, this indicates that refinement is beneficial for both welfare and scientific outcome in both sexes. Moreover, these experiments showed that sex biases in preclinical research due to perceived effects of the oestrous cycle is poorly justified.^8^, ^9^

Studying females would allow a greater diversity of diabetic phenotypes to be captured, ensuring representation of a more heterogenous clinical population. Indeed, significant sex differences in the prevalence of diabetes^45^, ^46^, ^47^, ^48^ and response to antidiabetic agents^49^ exists in humans. Consequently, there is increasing emphasis on the need to study females to improve translatability of research.^9^, ^10^, ^11^ Although refinement is important for animal welfare and the use of females is important for translatability, there is a common perception that subsequent lower glucose concentrations may preclude the ability to observe drug effects, impeding the predictive validity of the model. To challenge this, we further refined our GTT protocol by negating the need for glucose injection by training the mice to voluntarily ingest a glucose gel. Although this may not be a practical protocol for all experimental designs, we used it as proof‐of‐principle to show refinement is not to the detriment of detecting drugs effects. Indeed, we showed that voluntary ingestion of a glucose gel reduced the glucose peak when compared to gavage (which involves scruffing the mice). Although we cannot rule out the possibility of enhanced incretin effects with glucose gels due to chewing, the differences observed are also likely to be associated with the reduced stress of voluntary gel consumption.^28^, ^50^, ^51^ We then used this refined protocol (6‐hour fast, bedding retention at start of fast, voluntary ingestion of glucose gel) to test whether there was predictive validity in this model of GTT (ie, whether efficacy of clinically used drugs could be detected). Despite the lower glucose peaks seen in the controls, we were able to detect clear glucose‐lowering effects of metformin and exendin‐4 in both male and female mice using this most refined GTT. Furthermore, although differences between drug and control were smaller in female versus male mice, the variability of their responses was also reduced and, consequently, the sample sizes required to detect the effects of both drugs did not differ.

We have previously shown the impact of researcher intervention during different steps of the standard IP GTT which could be deemed unavoidable in standard laboratory settings (such as entering the room, measuring blood glucose concentrations by glucometer and administering the glucose by injection).^33^ In those studies, we estimated that researcher intervention during the IP GTT contributes to an increase in blood glucose concentrations of approximately 2 to 3 mM. In the present study, we focused on an aspect of the GTT that we felt could be easily refined in most laboratories: the fasting protocol. Indeed, this study highlights how even minor and seemingly inconsequential variations in the GTT fasting protocol can impact both blood glucose concentrations during the fast and glucose tolerance several hours later, even taking into consideration the unavoidable effects of researcher intervention associated with the start of the GTT (such as measuring blood glucose and administering glucose). Therefore, this study emphasizes the importance of experimental design on outcomes and hence the value of consistent procedures and full disclosure of methods. Moreover, we have refuted common misconceptions that lead to the exclusion of female mice, with females exhibiting less variable GTT responses which are not affected by the oestrous cycle, particularly when protocols are refined. Consequently, female mice were no less useful in detecting drug responses than male mice. Overall, this research also provides proof‐of‐concept in normoglycaemic mice to challenge the perception that a milder response to glucose challenge (whether due to refinement and/or sex of mice) impedes the ability to detect the effects of antidiabetic drugs. However, further research is required to ascertain whether these results are reproducible in different laboratories and mouse strains.

## 5 SUPPLEMENTARY INFORMATION

### 5.1 Surgical implantation of HD‐XG glucose telemetry devices and surgical recovery

Anaesthesia was induced in animals with 2.5‐5% isoflurane in 1 L/min 95% oxygen and maintained throughout surgery with 1.5‐2% in 0.5 L/min oxygen. Mice were administered 4 mg/kg subcutaneous carprofen (Carprieve; Centaur, UK) immediately prior to surgery and 24 hour post‐surgery. Mice were also administered 2 mg/kg Marcain (Centaur, UK) at the wound site following suturing. Mice were maintained at 37°C via a homeothermic heating blanket and rectal probe. The sensor portion of the probe was advanced towards the aortic arch, accessed via the left carotid artery, and sutured in place whilst the radio‐transmitter portion was advanced subcutaneously under the right abdominal flank along with ~0.5 ml saline resuscitation.^31^ Animals were recovered on their own in incubators overnight at 28°C after which they were transferred to the monitoring lab and reintroduced to their buddies.

### 5.2 Details of whole cage changes, bedding retention cage changes and no cage changes

Whole cage changing (WCC) consisted of the mice being placed in a new cage with new wood chippings, new bedding and new enrichment. Bedding retention cage changing (BRCC) consisted of mice being placed in a new cage with new wood chippings but the bedding and enrichment from the old cage was retained and placed in the new cage. No cage changing (NCC) consisted of mice not being handled and food being removed from the cage lid only.

### 5.3 Method for making the oral glucose and metformin gels for voluntary consumption

60% oral glucose gels were made using 80% (w/v) D‐glucose (Sigma‐Aldrich, UK) in distilled water. This was mixed with gelatine (Dr Oetker™) in water with sugar‐free chocolate flavouring (Nick's Stevia Drops™) in a 3:1 dilution. Mice weights from 24 hour prior to the GTT were used to make glucose gels to administer a dose of 2 g/kg.

Metformin gels were made using 0.18% (w/v) 1,1‐Dimethylbiguanide hydrochloride (Sigma‐Aldrich, UK) in distilled water. This was mixed with gelatine (Dr Oetker™) in water and sucralose sweetener (Splenda) in water with chocolate flavouring (Nick's Stevia drops™) in a 1:1:1 dilution. Sweetener control gels were made by mixing sucralose sweetener in water with chocolate flavouring and gelatine in water in a 2:1 dilution.

## FUNDING INFORMATION

This work was funded by the British Pharmacological Society through an AJ Clark studentship awarded to M.R.K. The British Heart Foundation (FS/20/8/34984) and the Royal Society (RGS\R2\192275) provided funding for the telemetry equipment.

## CONFLICT OF INTEREST

The authors declare no conflicts of interest.

### PEER REVIEW

The peer review history for this article is available at https://publons.com/publon/10.1111/dom.14811.

## Supporting information


**Figure S1.** Thirty‐six‐hour average 10‐second blood glucose concentrations following 6‐hour fast (commencing at 9:00 am) or 16‐hour fast (commencing at 5:00 pm) with bedding retention cage changes (BRCC) and subsequent intraperitoneal glucose tolerance tests (GTTs) for (A) males and (B) females. Black line = blood glucose concentrations following 6‐hour fast with BRCC; red line = blood glucose concentrations following 16‐hour fast with BRCC. Grey bar = dark phase. Data are mean ± SEM (n = 7 for males and females).

## Data Availability

Data available on request from the authors
