# Supplementary figures and images for: The glucose tolerance test in mice: Sex, drugs and protocol
Source: Diabetes Obes Metab. 2022 Jul 25;24(11):2241–52. doi: 10.1111/dom.14811 (PMC9795999; doi:10.1111/dom.14811)

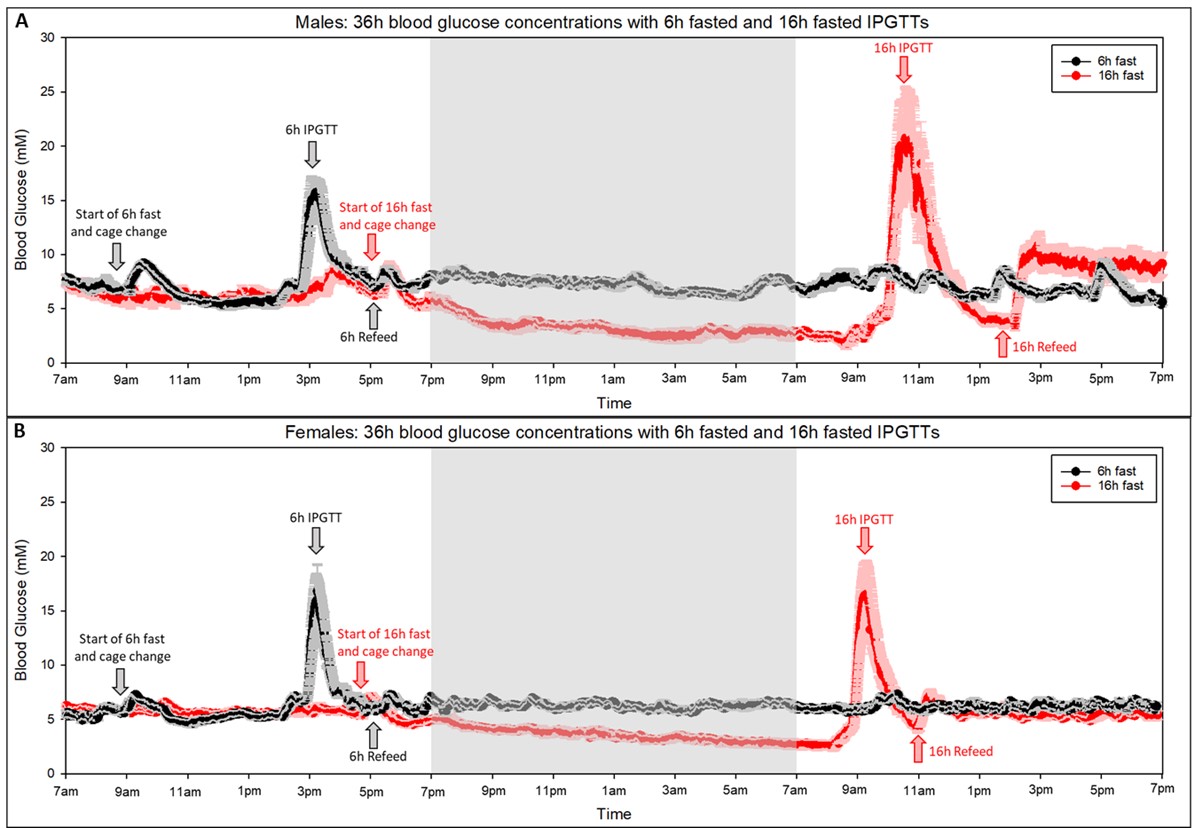

Supplement: Supplementary file 1 — Figure S1. Thirty‐six‐hour average 10‐second blood glucose concentrations following 6‐hour fast (commencing at 9:00 am) or 16‐hour fast (commencing at 5:00 pm) with bedding retention cage changes (BRCC) and subsequent intraperitoneal glucose tolerance tests (GTTs) for (A) males and (B) females. Black line = blood glucose concentrations following 6‐hour fast with BRCC; red line = blood glucose concentrations following 16‐hour fast with BRCC. Grey bar = dark phase. Data are mean ± SEM (n = 7 for males and females). [file DOM-24-2241-s001.jpg]
